# Outcomes and Its Associated Factors among Patients with Abdominal Trauma Requiring Laparotomy at Asella Referral and Teaching Hospital, South Central Ethiopia: A Retrospective Cross-Sectional Study

**DOI:** 10.1155/2024/5572633

**Published:** 2024-07-23

**Authors:** Wegene Tadesse Shenkutie, Taha Kaso, Abdene Weya Kaso, Gebi Agero

**Affiliations:** ^1^ Department of Surgery College of Health Science Arsi University, Asella, Ethiopia; ^2^ Department of Public Health College of Health Science Arsi University, Asella, Ethiopia

## Abstract

Trauma is a serious public health problem, and abdominal injuries are among the leading causes of hospitalization after trauma. Therefore, this study aimed to determine the outcome of abdominal trauma and its predictors in patients who underwent laparotomy at Asella Referral and Teaching Hospital (ARTH), South Central Ethiopia. We conducted a retrospective institutional based cross-sectional study of patients who underwent laparotomy for abdominal trauma at ARTH from October 1, 2015, to September 30, 2020. Bivariate and multivariate logistic regressions were used to determine associations between independent factors and mortality due to abdominal trauma, and a *P* value of <0.05 indicated statistical significance. Out of 139 patients, 110 (79.1%) were males and 88 (63.3%) aged <30 years old, with a mean age of 29 ± 15.73 years. The most common mechanism of injury was penetrating trauma, which accounted for 94 (67.6%) patients. The mortality rate was 21 (15.1%). Factors such as blunt mechanism of injury (95% CI: AOR: 3.36, 1.24–9.09), SBP < 90 mmHg at presentation (95% CI: AOR = 9.37, 3.28–26.80), time >6 hours from trauma to admission (95% CI: AOR: 5.44, 1.78–16.63), unstable intraoperative patient condition (95% CI: AOR = 8.82, 3.05–25.52), and patients who need blood transfusion (95% CI: AOR: 6.63, 1.92–22.91) were significantly associated with mortality. The mortality rate of abdominal trauma patients who underwent laparotomy was high. Therefore, healthcare providers should provide priority for traumatic patients as prolonged waiting time to get healthcare results in poor outcomes for the patients.

## 1. Introduction

Trauma is a health problem associated with physical injury and is the third leading cause of disability worldwide [1]. Globally, approximately five million people die annually from injuries, with around 90% of deaths occurring in low- and middle-income countries (LMICs) including Ethiopia [2, 3]. Globally, the abdomen is one of the most frequently injured areas in trauma patients which accounts for 25% of all traumatic events [4–6]. In Ethiopia, abdominal trauma is a health problem and accounts for approximately 9–14% of all trauma admissions and is the third cause of emergency laparotomy [3]. Abdominal injuries can be classified as blunt or penetrating, usually involving organs such as the spleen, intestines, stomach, liver, diaphragm, and kidneys [7, 8]. The majority of abdominal injuries are caused by blunt trauma secondary to Road Traffic Accident(RTA), while stab wounds and gunshot wounds are the most common types of penetrating injuries [9–13]. In most cases, penetrating abdominal injuries can be easily and reliably diagnosed, while blunt injury mechanisms can often be overlooked because clinical signs and symptoms are less obvious [13]. Thus, management of these injuries requires careful triage for appropriate intervention, although 25% of patients with abdominal trauma require surgery. The most common indication for laparotomy was hemodynamic instability or ongoing blood loss [14, 15]. Although abdominal trauma is a common indication for surgery, outcomes and complications after surgery vary from health facility to health facility. Previous epidemiological studies showed that postoperative complication rates range from 18% to 42%, while mortality ranges from 8.5% to 13% [7, 10, 11]. Sociodemographic factors, mechanism of injury, the presence of associated extraabdominal injuries, admission systolic blood pressure, time to injury to admission, length of hospital stay (LOS), anemia, and postoperative complications were reported to significantly predict mortality [10–12, 16–20]. Trauma victims have better outcomes in developed countries due to well-organized trauma care centers with multidisciplinary teams [21]. However, in LMICs including Ethiopia, trauma victims face catastrophic outcomes and complications due to inadequate and underdeveloped rescue systems and the lack of well-established response teams in the health facilities [21–24]. Even though few studies were conducted in Ethiopia among outcomes of abdominal trauma patients, there is variation in the magnitude of outcomes due to differences in the quality of healthcare and availability of trained trauma management teams. This poses difficulty for healthcare providers and leaders to make decisions regarding the quality of healthcare provided to abdominal trauma victims. Therefore, detailed evidence on the outcome of abdominal trauma among patients who had undergone laparotomy is needed to improve the quality of care for services provided to trauma patients. Therefore, this study aimed to assess the outcome of abdominal trauma and its predictors in patients who underwent laparotomy at Asella Referral and Teaching Hospital, South Central Ethiopia.

## 2. Methods and Materials

### 2.1. Study Setting and Period

The study was conducted at Asella Referral and Teaching Hospital (ARTH) from October 1 to 15, 2020. The hospital is located in Asella, a zonal town of the Arsi zone. Asella town is located 175 km from Addis Ababa, the capital of Ethiopia. There is one public health hospital and 3 health centers in Asella town administration. The hospital serves as a referral hospital for 9 hospitals in the Arsi zone. The hospital has a total of approximately 250 beds and more than 500 healthcare providers serving the community.

### 2.2. Study Design and Population

**Figure 1:**
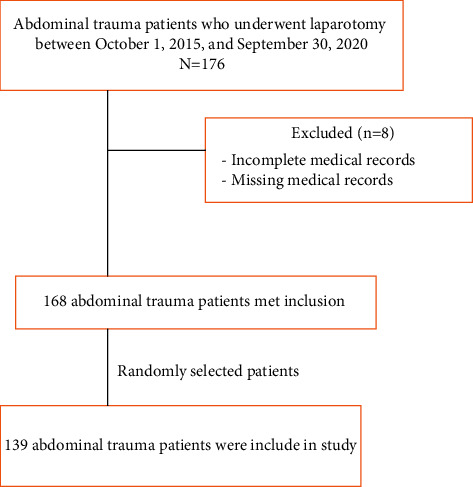
Flow diagram for study participant recruitment.

This was an institution-based cross-sectional study based on a retrospective medical record review over a period of five years. All patients who had abdominal trauma and underwent exploratory laparotomy for abdominal trauma at ARTH between October 1, 2015, and September 30, 2020, were source population. The study population included all randomly selected patients who underwent exploratory laparotomy for abdominal trauma at ARTH between October 1, 2015, and September 30, 2020. Abdominal trauma patients who underwent exploratory laparotomy and had incomplete information on medical charts were excluded (Figure 1).

### 2.3. Sample Size and Sampling Procedure

We estimated the sample size using the single population proportion formula ([*n* = [(*Zα*/2)2 *∗* *P*(1 − *P*)]/*d*2]). The study used the following assumption: 95% confidence level of *Zα*/2 = 1.96, 5% margin of error, and proportion of mortality after abdominal trauma laparotomy of around 9% from a study done in Addis Ababa [10] and 10% nonresponse rate. The final calculated sample size was 139 records of abdominal trauma patients. Finally, a systematic random sampling method was employed to review the medical records of the study participants.

### 2.4. Study Variables

Mortality after abdominal trauma laparotomy was the outcome variable, while sociodemographic factors (age, gender, and residence), mechanism of injury (penetrating, blunt, RTA, stab wound, or gunshot wound), Glasgow Coma Scale (GCS) score, associated injuries/extraabdominal injury, splenic injury, splenectomy, liver injury, blood pressure (SBP), blood transfusion, the time interval from incident to admission and from admission to intervention, patient condition during surgery, and length of hospitalization were independent variables.

### 2.5. Operational Definitions

Laparotomy: A vertical midline surgical incision through the abdominal wall that allows examination of an abdominal organ.

Death: The patient was admitted with abdominal trauma, underwent laparotomy, and died in hospital due to a trauma-related illness during treatment before discharge.

Alive: Patients admitted with abdominal trauma requiring laparotomy and started treatment regardless of cause and were discharged alive or cured.

Blood transfusion is a procedure in which whole blood or parts of blood products are transfused to a patient's bloodstream when hemoglobin <7 mg/dl [25, 26].

Blood products are any therapeutic substances derived from human blood for transfusion including whole blood, packed red blood cells, fresh frozen plasma, platelet concentrates, and cryoprecipitate [25, 26].

### 2.6. Data Collection Procedures and Quality Assurance

Data were collected from the medical records of patients who underwent surgery for abdominal trauma between October 1, 2015, and September 30, 2020, using a data abstraction form designed for this study. We extracted data such as sociodemographic variables, mechanism of injury, Glasgow Coma Scale score, associated injury/extraabdominal injury, splenic injury, splenectomy, liver injury, blood pressure, blood transfusion, the time interval from incident to admission and from admission to intervention, the status of the patient during the operation, the length of hospitalization, and the outcome of the laparotomy. Data were collected by trained medical students from the last years of medicine from the medical records of individual patients. The principal investigator supervised the overall data collection process and checked the completeness of the data daily.

### 2.7. Data Processing and Analysis

The collected data were checked for completeness and consistency. Data were cleaned, entered, processed, and analyzed using the Statistical Package for the Social Sciences (SPSS) version 25. Descriptive statistics such as frequencies and percentages were computed for categorical variables. Continuous variables were summarized using mean and standard deviation. Multivariate logistic regression analysis was performed to determine factors associated with mortality among abdominal trauma patients. All variables with a *P* value less than or equal to 0.25 in the bivariate logistic regression analysis were considered for the multivariate logistic regression model, and a *p* value ≤0.05 and an adjusted odds ratio (AOR) with 95% CI were used to declare the factors associated with the outcome variable. We checked multicollinearity using variable inflation while Hosmer and Lemeshow goodness-of-fit were computed to determine the fitness of the model.

### 2.8. Ethical Considerations

The Department of Surgery Research Ethics Review Board of Arsi University, College of Health Sciences, approved the study (Ref no.: A/CHS/RC//08/2020), and all methods were performed according to the relevant guidelines and regulations of the university. The Institutional Review Board of Arsi University waived oral informed consent because the study was conducted through patient chart review. Individual patients were not harmed, and the data were used only in this study.

## 3. Results

### 3.1. Sociodemographic Characteristics of the Study Participants

A total of 139 medical records were included in the study, 110 (79.1% were males, and 88 (63.3%) were aged ≤30 years old. The mean age of the participants was 29 + 15.73 years. A majority, 110 (79.1%), of the respondents lived in rural areas (Table 1).

### 3.2. Study Participants' Clinical Characteristics at the Time of Presentation

More than three-fifths (67.6%) of the participants were operated for penetrating mechanisms of injuries. Out of 45 (32.4%) blunt injuries, 25 (55.6%) occurred due to RTA, while 44.4% were related to other types of accidents such as falls, animal kicks, and assault and direct blow injuries. In this study, 93 (66.9%) of patients were presented to the hospital within 6 hours of trauma. Regarding vital signs at presentation, 83 (59.7%) had a systolic blood pressure (SBP) ≥90 mmHg, 83 (59.7%) had a hematocrit greater than 30%, and 74 (53.2%) had a heart rate less than 100 (Table 2).

### 3.3. Clinical Characteristics of the Study Participants during the Intraoperative Phase

**Figure 2:**
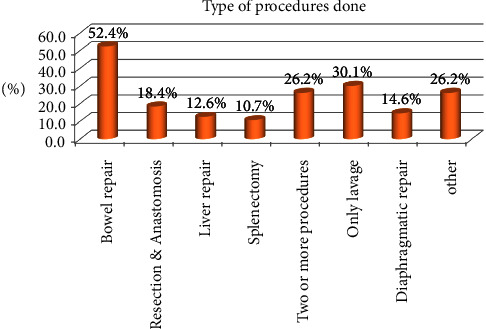
Procedure performed according to the organ injured.

Out of 139 patients, 113 (81.3%) had an organ (solid or hollow viscus) injury. The magnitude of negative laparotomies was 16 (11.5%). In this study, the small bowel was the most commonly injured organ, 21 (51.2%) in blunt injuries' patients, followed by the splenic, 14 (34.2%) and liver, and 10 (24.4%) organ injuries. In addition, the small intestine 30 (41.7%) was the most frequently injured organ in patients with penetrating injuries (Table 3). Of the 139 patients, 103 (74.1%) underwent hollow or solid organ repair, while 31 (30.1%) patients underwent lavage. Of the total number of cases, bowel repair was performed in 54 (52.4%), 19 (18.4%) underwent resection and anastomosis, 15 (14.6%) underwent diaphragm repair, 13 (12.6%) underwent liver repair, and 11 (10.7%) underwent splenectomy (Figure 2).

### 3.4. Patient's Postoperative Clinical Conditions and Outcomes

**Figure 3:**
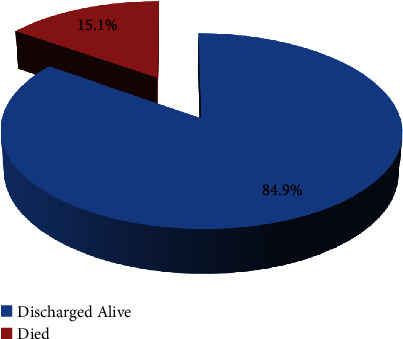
Outcome of abdominal trauma for laparotomy at ATRH, South Central Ethiopia, 2020.

Of the 139 operated patients with abdominal trauma, 17 (12.2%) were admitted to the ICU, 43 (30.9%) required blood transfusion, and 29 (20.9%) were in serious or critical condition. The postoperative complication rate was 31 (22.3%). The most common postoperative complication was surgical site infection (SSI) in 13 (41.9%) patients. Other complications were respiratory complications in 7 (22.6%) patients and irreversible shock in 12 (38.7%) patients. The median length of hospital stay for patients who underwent laparotomy was 6 days (range, 3 hours to 40 days) (Table 4). The hospital mortality rate was 21 (15.1%), while 118 (84.9%) patients were discharged home alive (Figure 3).

### 3.5. Factors Associated with Mortality due to Abdominal Trauma

In the binary logistic regression analysis, variables such as time to trauma to admission, mechanism of injury, SBP <90 mmHg at presentation, presence of associated extraabdominal injury, liver injury, spleen injury, need for blood transfusion, intraoperative patient condition, and time to admission to operation were statistically associated with mortality due to abdominal trauma (*p* ≤ 0.25). However, after adjusting for confounding variables such as time to trauma to admission, blunt mechanism of injury, SBP <90 mmHg at presentation, need for blood transfusion, and intraoperative patient condition showed statistically significant association with mortality due to abdominal trauma. This study showed that respondents who sustained a blunt mechanism of injury were nearly three times more likely to die than those who sustained a penetrating injury [AOR = 3.36; 95% CI, 1.24–9.09]. Patients who had SBP <90 mmHg at presentation had nearly nine times the odds of death than their counterparts [AOR = 9.37; 95% CI, 3.28, 26.8]. In addition, the odds of death were 5.44 times higher in patients who had a delay of ≥six hours to reach the hospital after trauma than in their counterparts [AOR = 5.44; 95% CI, 1.78–16.63]. Moreover, patients who had unstable intraoperative conditions were almost nine times more likely to die than patients who had stable intraoperative conditions [AOR = 8.82; 95% CI = 3.05–25.52]. The probability of death after abdominal trauma was 6.63 times higher among patients who needed blood transfusion after operation than their counterparts [AOR = 6.63; 95% CI, 1.92–22.91] (Table 5).

## 4. Discussion

Trauma still accounts for a significant number of emergency visits worldwide. Abdominal injuries contribute significantly to the morbidity and mortality of trauma patients in most public health facilities [27, 28]. In this study, the mortality rate for abdominal trauma laparotomy was 15.1% (95% CI: 9.0–21%), which is similar to studies conducted in the Netherlands (16.7%) [29], northern Tanzania (13.2%) [12], and northern Uganda (15.6%) [30]. However, these findings are higher than those of studies conducted in Germany (5.1%) [31], Egypt (7.5%) [13], and Addis Ababa, Ethiopia (8.5%) [10]. The most probable conditions for the discrepancy might be due to variations in the level of setting, accessibility of the healthcare facility, study period, availability of blood/blood products, and trauma management team in the health facilities.

In this study, patients who sustained blunt injury mechanisms were nearly eight times more likely to die than those who sustained penetrating injuries. This finding is supported by previous studies conducted by Joseph et al. [18] and Addis Ababa [10]. This could be the result of associated extraabdominal injuries, which are more common in blunt than in penetrating mechanisms of injury. Patients with blunt abdominal trauma arrived late because there was no outwardly visible trauma, complicating treatment. Another factor found to be significantly associated with abdominal trauma laparotomy mortality was SBP <90 mmHg at presentation. This finding is consistent with the findings of previous studies from Tanzania [12] and Addis Ababa [10]. This can be caused by bleeding that leads to decomposition before the patient arrives at the hospital.

The study revealed that patients who were unstable during the intraoperative phase were nearly nine times more likely to die than those who were stable during surgery. This is in line with a study conducted in Tanzania [12] and Eastern Ethiopia [32], which found a higher mortality rate in severely injured patients. In addition, the study found higher odds of death in patients who had delayed ≥6 hours to reach the hospital after trauma than in their counterparts. This finding is supported by findings from a study done in Tanzania [12] and Dilla, South Ethiopia [33]. This could be because severely injured patients who are delayed in utilizing healthcare services on time might face severe bleeding due to a lack of prehospital care which can lead to death [34]. Moreover, this study found a higher probability of death after abdominal trauma among respondents who needed a blood transfusion after an operation than their counterparts. This finding is in line with the study done at Jimma University Specialized Hospital, in southwestern Ethiopia [35]. This can be explained by the fact that abdominal trauma patients who need blood transfusion might have severe bleeding due to traumatic hemorrhage or a major surgical procedure performed to manage multiple organs damaged that require massive blood transfusion [36–38]. However, the lack of a well-organized miniblood bank with sufficient blood products to manage severe blood loss that resulted in platelet dysfunction and coagulopathy due to consumption through massive blood transfusion may have contributed to the poor outcomes of these patients. Although our study is the first to provide insight into the outcome of abdominal trauma laparotomy at the Asella Referral and Teaching Hospital, it has several limitations. First, this study was retrospective, and there was a lack of information on patient characteristics and clinical conditions at presentation and during and after surgery. Second, the study was limited by its single-center nature and small sample size, which affected the analysis of factors associated with the outcome. Third, the study included patients only up to the day of discharge, undermining the mortality rate.

## 5. Conclusion

In this study, penetrating abdominal injury was the most common type of injury. The proportion of mortality from abdominal trauma among patients who underwent laparotomy was high. Blunt mechanism of injury, SBP <90 mmHg, patient condition during surgery, time ≥6 hours from trauma to admission, and requiring blood transfusion were associated with mortality. Therefore, a health facility should organize a miniblood bank with sufficient blood and blood products in a way that can provide massive blood transfusion at full capacity to reduce mortality due to abdominal trauma. Besides, healthcare providers should provide priority for traumatic patients as the prolonged waiting time to obtain healthcare service results in poor outcomes for patients.

## Figures and Tables

**Table 1:** Sociodemographic characteristics of abdominal trauma patients at ARTH, South Central Ethiopia, 2020.

Variable	Categories	Frequency (%)
Sex	Male	110 (79.1)
	Female	29 (20.9)

Age category	≤30	88 (63.3)
	>30 years	51 (36.7)
	Mean ± SD	29 ± 15.73

Residence	Urban	29 (20.9)
	Rural	110 (79.1)

Marital status	Single	51 (36.7)
	Married	54 (38.8)
	Unknown	34 (24.5)

**Table 2:** Clinical characteristics of abdominal injury patients at the time of presentation at ARTH, South Central Ethiopia, 2020.

Clinical characteristics	Categories	Frequency (%)
Mechanism of injury	Penetrating	94 (67.6)
	Blunt	45 (32.4)

Type of penetrating	Stab	55 (58.5)
	Gunshot	18 (19.1)
	Cattle horn	12 (12.5)
	Others	9 (9.4)

Type of blunt	RTA	25 (55.6)
	Others	20 (44.4)

Time from trauma to admission	<6 hrs	93 (66.9)
	6–24 hrs	31 (22.3)
	>24 hrs	15 (10.8)

Pulse rate at presentation	60–100	74 (53.2)
	Not palpable or >100	65 (46.8)

SBP in mmHg	<90	56 (40.3)
	≥90	83 (59.7)

Associated extraabdominal injury	No	90 (64.7)
	Yes	49 (35.3)

Type of associated extraabdominal injuries	Chest injury	24 (49)
	Fracture	10 (20.4)
	Other	15 (29.6)

Hematocrit	<21	2 (1.4)
	21–30	54 (38.9)
	>30	83 (59.7)

Time from admission to operation	≤2 hrs	81 (58.3)
	>2 hrs	58 (41.7)

**Table 3:** Organ injuries depending on the mechanism of injury of trauma patients treated with laparotomy at ARTH, Asella, 2020.

		Mechanism of injury	
		Penetrating	Blunt
Organ injured	Splenic injury	5 (6.9%)	10 (24.4%)
	Liver injury	19 (26.4%)	10 (24.4%)
	Small bowel injury	30 (41.7%)	21 (51.2%)
	Colonic injury	24 (33.3%)	4 (9.8%)
	Stomach injury	12 (16.7%)	1 (2.4%)
	Diaphragmatic injury	15 (20.8%)	1 (2.4%)
	Other^a^	13 (18.1%)	5 (12.2%)
Total		72 (63.7%)	41 (36.3%)

*Note.* ^a^Kidney injury, duodenal injury, bladder injury, vascular injury, and retroperitoneal hematoma.

**Table 4:** Postoperative clinical conditions of abdominal trauma patients at ARTH, South Central Ethiopia, 2020.

Clinical characteristics	Categories	Frequency (%)
ICU admission	No	122 (87.8)
	Yes	17 (12.2)

Need blood transfusion	No	96 (69.1)
	Yes	43 (30.9)

Postoperative progress	Smooth	110 (79.1)
	Critical/serious	29 (20.9)

Complication	No	108 (77.7)
	Yes	31 (22.3)

Type of complication	SSI	13 (9.1)
	Respiratory complication	7 (5.0)
	Irreversible shock	12 (8.6)
	Other	5 (3.6)

Relaparotomy	No	129 (92.8)
	Yes	10 (7.2)

Indication for relaparotomy	Postoperative collection and/or leak	5 (50.0)
	Dehiscence	1 (10.0)
	Second look	4 (40.0)

**Table 5:** Multivariate logistic regression analyses of factors associated with mortality due to abdominal trauma in ARTH patients, South Central Ethiopia, 2020.

Characteristics	Dead (*n*)	Alive (*n*)	COR (95% CI)	AOR (95% CI)
Age				
≤30	16 (18.2)	72 (81.8)	1.41 (0.48–4.13)	
>30	5 (9.8)	46 (90.2)	1	
Gender				
Female	4 (13.8)	25 (86.2)	1	
Male	17 (15.5)	93 (84.5)	1.14 (0.35–3.70)	
Mechanism of injury				
Penetrating	10 (10.6)	84 (89.4)	1	1
Blunt	11 (24.4)	34 (75.6)	2.96 (1.15–7.63)	3.36 (1.24–9.09)^*∗*^
Time from trauma to admission				
≤6 hours	8 (8.6)	85 (91.4)	1	1
>6 hours	13 (28.3)	33 (71.7)	4.01 (1.53–10.56)	5.44 (1.78–16.63)^*∗*^
SBP (admission)				
<90 mmHg	16 (28.6)	40 (71.4)	6.24 (2.13–18.27)	9.37 (3.28–26.80)^*∗*^
≥90 mmHg	5 (6.0)	78 (94.0)	1	1
Associated injury				
No	9 (10.0)	81 (90.0)	1	1
Yes	12 (19.6)	37 (80.4)	2.92 (1.31–7.53)	1.93 (0.69–5.39)
Time to admission to operation				
≤2 hours	7 (8.6)	74 (91.4)	1	1
>2 hours	14 (24.1)	44 (75.9)	3.36 (1.26–8.97)	3.15 (0.98–11.12)
Liver injury				
No	10 (9.1)	100 (90.9)	1	1
Yes	11 (37.9)	18 (62.1)	6.11 (2.27–16.49)	3.81 (0.95–15.22)
Spleen injury				
No	14 (11.3)	110 (88.7)	1	1
Yes	7 (46.7)	8 (53.3)	6.88 (2.16–21.86)	3.64 (0.91–14.54)
Intraoperatively patient condition				
Stable	9 (8.0)	104 (92.0)	1	1
Unstable	12 (46.2)	14 (53.8)	9.91 (3.54–27.71)	8.82 (3.05–25.52)^*∗*^
Needed blood transfusion				
No	5 (5.2)	91 (94.8)	1	1
Yes	16 (37.2)	27 (62.8)	10.78 (3.6–32.15)	6.63 (1.92–22.91)^*∗*^

^*∗*^, *p* < 0.05, COR: crude odds ratio, AOR: adjusted odds ratio.

## Data Availability

Data used or analyzed during this study are available from the corresponding author upon reasonable request.

## Abbreviations

AOR: Adjusted odds ratio
ATLS: Advanced trauma life support
BP: Blood pressure
CI: Confidence interval
COR: Crude odds ratio
FAST: Focused assessment with sonography for trauma
GCS: Glasgow Coma Scale
LOS: Length of stay
OR: Odds ratio
PR: Pulse rate
PSOR: Saturation of oxygen
RR: Respiratory rate
RTA: Road traffic accident
SBP: Systolic blood pressure
SD: Standard deviation
